# Fluid Biomarkers in Optical Coherence Tomography for Visual Outcome in Polypoidal Choroidal Vasculopathy

**DOI:** 10.3390/jpm14060574

**Published:** 2024-05-27

**Authors:** I-Hsin Ma, Tso-Ting Lai, Chang-Hao Yang, Tzyy-Chang Ho, Chung-May Yang, Yi-Ting Hsieh

**Affiliations:** 1 Department of Ophthalmology, National Taiwan University Hospital, Taipei 100225, Taiwan; nuitdedecembre@gmail.com (I.-H.M.); b91401005@ntu.edu.tw (T.-T.L.); chyangoph@ntu.edu.tw (C.-H.Y.); hotchang@ntu.edu.tw (T.-C.H.); chungmay@ntu.edu.tw (C.-M.Y.); 2 Department of Ophthalmology, National Taiwan University Hospital Hsin-Chu Branch, Biomedical Park Hospital, Hsinchu 302058, Taiwan; 3 Department of Ophthalmology, College of Medicine, National Taiwan University, Taipei 100229, Taiwan

**Keywords:** optical coherence tomography, polypoidal choroidal vasculopathy

## Abstract

**Purpose:** To investigate the associations between fluid accumulation at different levels in the retina and visual outcome in polypoidal choroidal vasculopathy (PCV). **Design:** A retrospective observational study. Institutional setting. **Study Population:** A total of 91 eyes from 91 patients of PCV were included, with 65 receiving intravitreal aflibercept monotherapy and 26 receiving combined intravitreal ranibizumab and photodynamic therapy (PDT). Observation Procedures: Best-corrected visual acuity (BCVA) and optical coherence tomography (OCT) examination results were recorded at baseline and 3, 6, and 12 months after treatment. **Main Outcome Measures:** The correlations between visual outcomes and fluid biomarkers including intraretinal fluid (IRF), subretinal fluid (SRF), serous pigment epithelium detachment (PED), and hemorrhage at fovea were analyzed. **Results:** No differences in treatment outcomes were noted between patients receiving aflibercept and those receiving combined ranibizumab and PDT. IRF and hemorrhage at baseline predicted poorer vision at 3, 6, and 12 months. The presence of IRF was associated with poorer vision at 6 months and 12 months (*p* < 0.05 for all). The presence of SRF or PED was not associated with better vision at any time point. No differences in the correlations between fluid markers and visual outcomes were noted between thin and thick subfoveal choroidal thickness groups. **Conclusions:** For PCV, IRF and hemorrhage at baseline served as surrogates for poor visual prognosis after treatment, and IRF was a biomarker for poor vision during the treatment course. No fluid markers predicted good visual prognosis or had a positive impact on vision at any time point.

## 1. Introduction

Polypoidal choroidal vasculopathy (PCV) is distinguished by the presence of dilated choroidal vasculature and neovascular complexes, characterized by polypoidal or aneurysmal terminations, and occasionally branched vascular networks situated beneath the retinal pigment epithelium (RPE) [1]. Visual impairment results when there is fluid leakage or hemorrhage due to PCV. Fluid accumulation or hemorrhage may occur at different anatomical layers, including the intraretinal, subretinal, or sub-RPE layers [2]. Optical coherence tomography (OCT) serves as a valuable imaging modality, facilitating the precise delineation of abnormal substance accumulation within these distinct layers.

PCV demographics differs among ethnicities, in Asian population, the lesions are mainly peripapillary or located in macula. Resultant serosanguinous macular detachment is the main cause for vision decrease. Reported mean visual acuity prior to treatment was around logMAR 0.4~0.5, and an improvement of 0.2 logMAR after treatment could be achieved [3,4,5]. Moreover, the prevalence of PCV in Asian populations differs significantly from that in White populations, with 23–62% of presumed AMD cases meeting PCV criteria among Asians, compared to only 4–9.8% in White populations [6,7,8].

With PCV being very prevalent and potentially leading to drastic visual debilitation, substantial research was performed on finding the most suitable treatment for PCV. Photodynamic therapy (PDT) was once the mainstay of PCV therapy for many years. Nevertheless, multiple studies advocate therapy with injections of anti-vascular endothelial growth factor (anti-VEGF) [9,10,11,12,13,14]. Treatment with anti-VEGF therapy alone is effective in eliminating the fluid from macular area and improving visual acuity; however, it is less effective against closing the polypoidal lesions [15,16,17]. Combination therapy, with various combination protocols, has also been suggested by different research groups [10,16,18,19,20,21,22,23]. Our group has also published the visual prognosis between two different treatment protocols, with similar outcomes. In this study, a difference was found, with better visual prognosis in non-pachychoroid patients treated with aflibercept monotherapy; however, this was not an universal finding as compared with the literature [24]. Crucial indicators of treatment efficacy encompass the resolution of fluid and hemorrhage, pivotal in gauging disease activity in both polypoidal choroidal vasculopathy (PCV) and neovascular age-related macular degeneration (nAMD) [18,25,26,27]. Recurrent hemorrhage is regarded as a poor prognostic sign for visual outcome in PCV [28], while the reappearance of fluid may signal the necessity for intensified therapeutic interventions [29,30]. The overarching goal of all treatment modalities is to stabilize the underlying pathology and facilitate improved visual outcomes. Nevertheless, the existing literature suggests that the impact of fluid accumulation in nAMD is multifaceted, indicative of the intricate pathophysiological mechanisms underlying this condition. Extensive exploration into the differential effects of fluid accumulation in nAMD remains imperative for elucidating optimal treatment strategies and enhancing clinical management practices. Post hoc analysis from the comparison of age-related macular degeneration treatment trials (CATT) study reported protective effects of persistent subretinal fluid (SRF), while intraretinal fluid (IRF) accounted for poor visual acuity (VA) in nAMD [31]. As for sub-RPE fluid, subgroup analysis from the phase III, double-masked, multicenter, randomized, active treatment-controlled study of the efficacy and safety of 0.5 mg and 2.0 mg Ranibizumab administered monthly or on an as-needed basis (PRN) to patients with subfoveal neovascular age-related macular degeneration (HARBOUR) reported that sub-RPE fluid had no adverse effects on VA gain. However, some real-world studies have reported that sub-RPE fluid is associated with worse vision [32,33,34]. To our knowledge, the effects of fluid on VA in PCV have not been well studied. In this study, we evaluated the effects of fluid in different layers on VA and prognosis in PCV.

## 2. Methods

Patients diagnosed with PCV in National Taiwan University Hospital (NTUH) between 2015 and 2018 were retrospectively recruited. Diagnostic criteria of PCV included a branching network of vessels in the inner choroid, and vascular dilations at the border of the network of vessels in indocyanine green angiography (ICGA), according to Spaide and Yannuzzi [35]. The condition could couple with subretinal hemorrhage, sub-RPE hemorrhage, and/or macular edema documented by fundus photography or Optovue Avanti RTVue XR OCT (Optovue, Inc., Fremont, CA, USA). Non-ICGA criteria including sub-RPE ring-like structure on cross-sectional OCT, complex RPE elevation on en-face OCT, and sharp-peaked PED on cross-sectional OCT were also used as features for aiding PCV diagnosis. Two retina specialists reviewed the exams independently and confirmed the diagnoses according to the consensus of the Asia–Pacific Ocular Imaging Society PCV Workgroup [36]. Patients with proliferative diabetic retinopathy, myopic maculopathy, previous rhegmatogenous retinal detachment, or other causes of choroidal neovascularization were excluded. If both eyes of a patient meet the inclusion criteria, only the results from the eye that received treatment first were included. The study was approved by the institutional review board of NTUH (No.: 202111070RIND) and followed the tenets of the Declaration of Helsinki. A waiver of informed consent was obtained due to its retrospective nature.

Demographic data, best-corrected visual acuity (BCVA) in terms of the logarithm of the minimum angle of resolution (LogMAR), and OCT biomarkers were recorded at initial presentation, and at 3, 6, and 12 months after initial treatment. OCT was performed under mydriatic examination in all patients. OCT biomarkers included IRF, SRF, and serous pigment epithelium detachment (PED) involving the fovea. Subfoveal choroidal thickness (SFCT) was measured on OCT and 267.5 µm was used for thickness stratification [26]. Subretinal or subRPE hemorrhage involving the fovea observed by indirect ophthalmoscopy or fundus photography was also recorded. The treatment modality and number of anti-VEGF injections during the 12-month period were documented. Treatment given was based on National Health Insurance (NHI) policy at the time and based on mutual physician and patient agreement. Patients were on different treatment protocols during that period, first was the photodynamic therapy (PDT) plus ranibizumab group, the other was the intravitreal aflibercept (IVA) injection alone group. The reason for this distinction depended on the reimbursement program chosen then. In the period, patients applied for ranibizumab treatment which was granted for one PDT session.

Protocols for these two treatments were as follows: In the combination treatment group, patients received an initial intravitreal ranibizumab (IVR) injection of 0.5 mg per eye, closely followed by standard PDT (verteporfin: 6 mg/m^2^; full laser irradiance: 600 mW/cm^2^; treatment time: 83 s), covering all branching vascular network and polyps within 1 week. The second injection of ranibizumab was administered 1 month after the initial IVR, and there was another 1 month separation before the third dosage of intravitreal Ranibizumab injection. Additional anti-VEGF injections were continued at a pro re nata basis (with intervals of ≥28 days for each injection) according to persistent disease activity during follow-ups. Rescue therapy with PDT was performed only in patients with persistent or newly developed polypoidal lesions plus disease activity, with an interval of ≥3 months from the previous PDT. In the intravitreal aflibercept (IVA) injection alone group, three monthly injections followed by pro re nata injection with an interval of ≥28 days was designed.

### Statistical Analysis

LogMAR of the BCVA at baseline and after treatment was analyzed, stratified by the presence of different types of fluid. Normality check was performed with Shapiro–Wilk test. Student *t*-test and Mann–Whitney U test were used to examine the differences in VA (logMAR) among the different groups. The statistical analysis was performed using SPSS software (SPSS 22.0; SPSS Inc., Chicago, IL, USA) and statistical significance was set at *p* < 0.05. Multiple regression analysis used Bonferroni correction to control false discovery rate, and significance was set at *p* < 0.01 after correction.

## 3. Results

### 3.1. Demographic Data and Treatment Protocols

**Figure 1:**
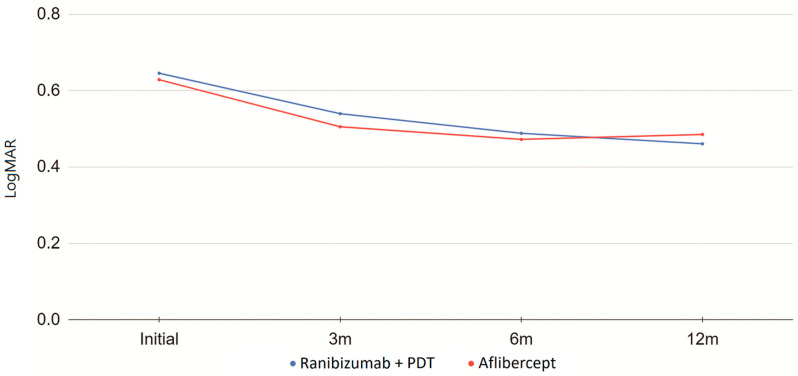
Visual acuity presented in logMAR at different time points before and after treatment in patients according to different treatment protocols. (3 m: 3 months post-treatment, 6 m: 6 months post-treatment, 12 m: 12 months post-treatment.) Blue line: combined ranibizumab and photodynamic therapy. Red line: aflibercept monotherapy.

A total of 91 eyes from 91 patients (57 men and 34 women) were included for analysis. The mean age of the subjects was 64.5 ± 9.8 years. Of the 91 eyes, 65 received intravitreal aflibercept monotherapy and 26 were treated with combined intravitreal ranibizumab and PDT therapy. After treatment, the logMAR of the BCVA improved from 0.63 ± 0.43 at baseline to 0.48 ± 0.45 at 12 months (*p* < 0.001). For the aflibercept monotherapy group, the number of injections within 12 months was 3.52 ± 3.60. For the therapy group that received ranibizumab plus PDT, the number of injections was 3.08 ± 4.79, and the number of PDT sessions was 1.42 ± 0.76 within 12 months. The vision of both groups improved after treatment, and no differences in BCVA were observed between the two treatment groups at any time point (Figure 1).

### 3.2. Intraretinal Fluid

**Figure 2:**
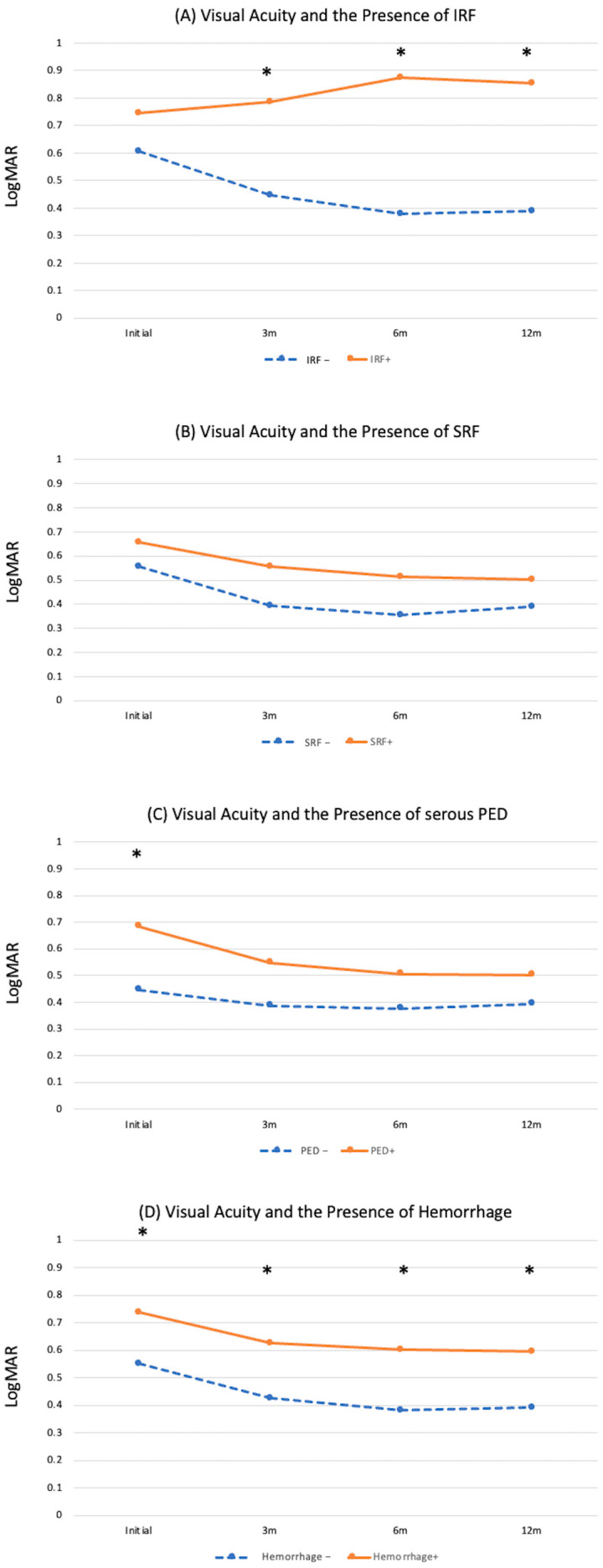
Visual acuity presented in logMAR at different time points before and after treatment in patients according to the presence of fluid biomarkers at baseline. (3 m: 3 months post-treatment, 6 m: 6 months post-treatment, 12 m: 12 months post-treatment.) (**A**) Intraretinal fluid (IRF), (**B**) subretinal fluid (SRF), (**C**) serous pigment epithelial detachment (PED). (**D**) Subretinal or sub-RPE hemorrhage. Asterisks denotes statistical significance with *p* < 0.05.

At baseline, 18 eyes (19.8%) presented with IRF and 73 eyes (80.2%) presented without IRF, and the logMAR of the BCVA were 0.74 ± 0.40 and 0.60 ± 0.43, respectively (*p* = 0.27). After treatment, the vision of eyes with IRF at baseline did not improve at all (logMAR of the BCVA = 0.78 ± 0.54, 0.87 ± 0.67, and 0.85 ± 0.60 at 3, 6, and 12 months, respectively), while the vision of eyes without IRF at baseline improved to 0.45 ± 0.36, 0.38 ± 0.37, and 0.39 ± 0.36 at 3, 6, and 12 months, respectively. Vision was significantly different between the two groups at 3, 6, and 12 months (*p* < 0.001 for all; Figure 2A).

**Figure 3:**
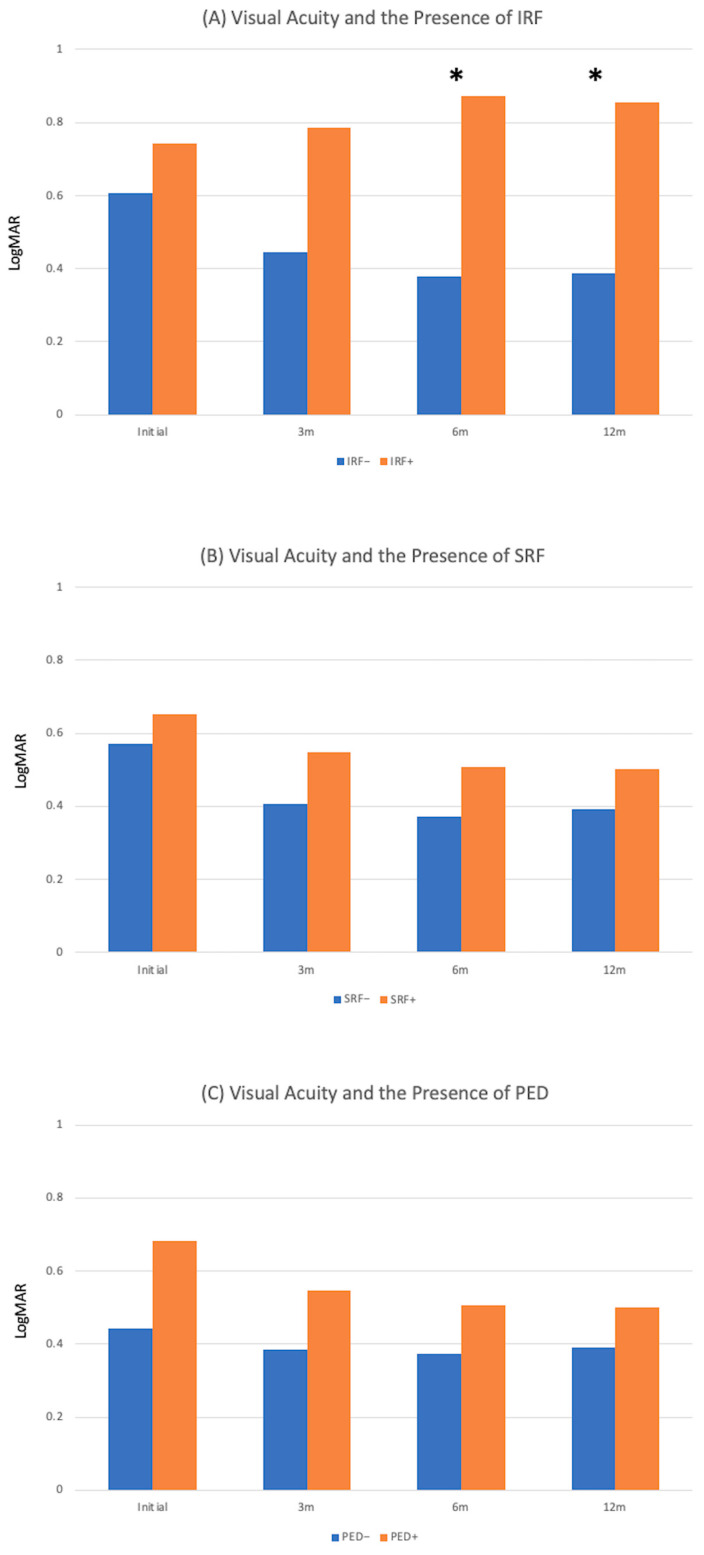
The relationship between visual acuity (LogMAR) and the status of fluids in different layers and at different time points (3 m: 3 months post-treatment, 6 m: 6 months post-treatment, 12 m: 12 months post-treatment). (**A**) Intraretinal fluid (IRF), (**B**) subretinal fluid (SRF), (**C**) serous pigment epithelial detachment (PED). Asterisks denotes statistical significance with *p* < 0.05.

After treatment, the proportions of eyes with IRF decreased from 19.8% at baseline to 8.8%, 13.2%, and 15.4% at 3, 6, and 12 months, respectively. Figure 3A shows that eyes with IRF had poorer vision than those without IRF at 6 and 12 months (*p* < 0.05 for both).

### 3.3. Subretinal Fluid

Prior to treatment, 69 eyes (75.8%) presented with SRF and the other 22 (24.2%) were free of SRF. The logMAR of the BCVA at baseline were 0.65 ± 0.42 and 0.57 ± 0.47, respectively (*p* = 0.39). After treatment, the logMAR of the BCVA of those with SRF at baseline improved to 0.55 ± 0.44, 0.51 ± 0.50, and 0.50 ± 0.47 at 3, 6, and 12 months, respectively. The logMAR of the BCVA of those without SRF at baseline improved to 0.41 ± 0.37, 0.37 ± 0.4, and 0.39 ± 0.41, respectively. VA improved after treatment in both groups, and no differences in the BCVA were observed between the two groups at any time point (Figure 2B). 

After treatment, the proportions of eyes with SRF decreased from 75.8% at baseline to 20.9%, 25.3%, and 31.9% at 3, 6, and 12 months, respectively. Figure 3B shows a trend for the vision of eyes with SRF to be worse than eyes without SRF, although there were no significant differences between the two groups any time point.

### 3.4. Serous Pigment Epithelial Detachment

Serous PED was present in 72 eyes (79.1%) at baseline with a logMAR of the BCVA of 0.68 ± 0.45, which was worse than the vision of eyes without serous PED at baseline (0.45 ± 0.25, *p* = 0.02). The vision of both groups improved after treatment, and no significant differences in vision between the two groups were observed during the follow-up period (Figure 2C). 

After treatment, the proportion of serous PED decreased from 76.9%, at baseline to 49.5%, 40.7% and 38.5% at 3, 6, and 12 months, respectively. The vision of eyes with serous PED had a worse trend than those without, although the differences between the two groups were only significant at baseline (*p* = 0.02; Figure 3C).

### 3.5. Subretinal/subRPE Hemorrhage

Out of the 91 eyes, 39 (42.9%) had subretinal or subRPE hemorrhage involving the fovea at initial presentation. The logMAR of the BCVA of these eyes at baseline was 0.74 ± 0.52, which was significantly worse than those without subfoveal hemorrhage (0.55 ± 0.33, *p* = 0.04). Although VA improved in both groups after treatment, those with subfoveal hemorrhage at baseline still had worse vision throughout the follow-up periods (*p* < 0.05 for all; Figure 2D).

### 3.6. Effect of Subfoveal Choroidal Thickness

**Figure 4:**
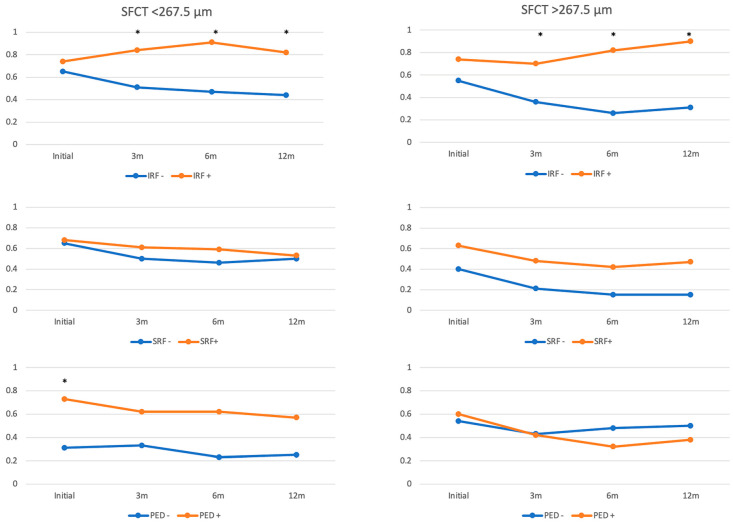
Visual acuity presented in logMAR at different time points before and after treatment according to the presence of fluid biomarkers at baseline in patients with different subfoveal choroidal thickness. (SFCT: subfoveal choroidal thickness, 3 m: 3 months post-treatment, 6 m: 6 months post-treatment, 12 m: 12 months post-treatment, IRF: intraretinal fluid, SRF: Subretinal fluid, PED: Serous pigment epithelial detachment). Asterisks denotes statistical significance with *p* < 0.05.

The possible confounding effect of SFCT on fluid biomarkers was evaluated by using subgroup analysis for all layers of fluid. Both thin and thick SFCT subgroups showed worse vision in patients with initial presence of IRF, and the statistical significance was achieved during all follow up time points (*p* < 0.05), which was compatible with the combined data. For SRF and PED, the subgrouping data was also similar to the combined data, and no paradoxical result was found (Figure 4).

### 3.7. Multiple Regression Analysis for Factors Associated with Visual Prognosis at 12 Months

Poor initial BCVA and the presence of IRF were correlated with worse VA at 12 months post-treatment (*p* < 0.001). Other factors such as age, the presence of SRF, sPED, and subfoveal hemorrhage were not significantly associated with poor VA when analyzed with multiple regression. 

### 3.8. Fluid Biomarkers at Three Months for Visual Prognosis

**Figure 5:**
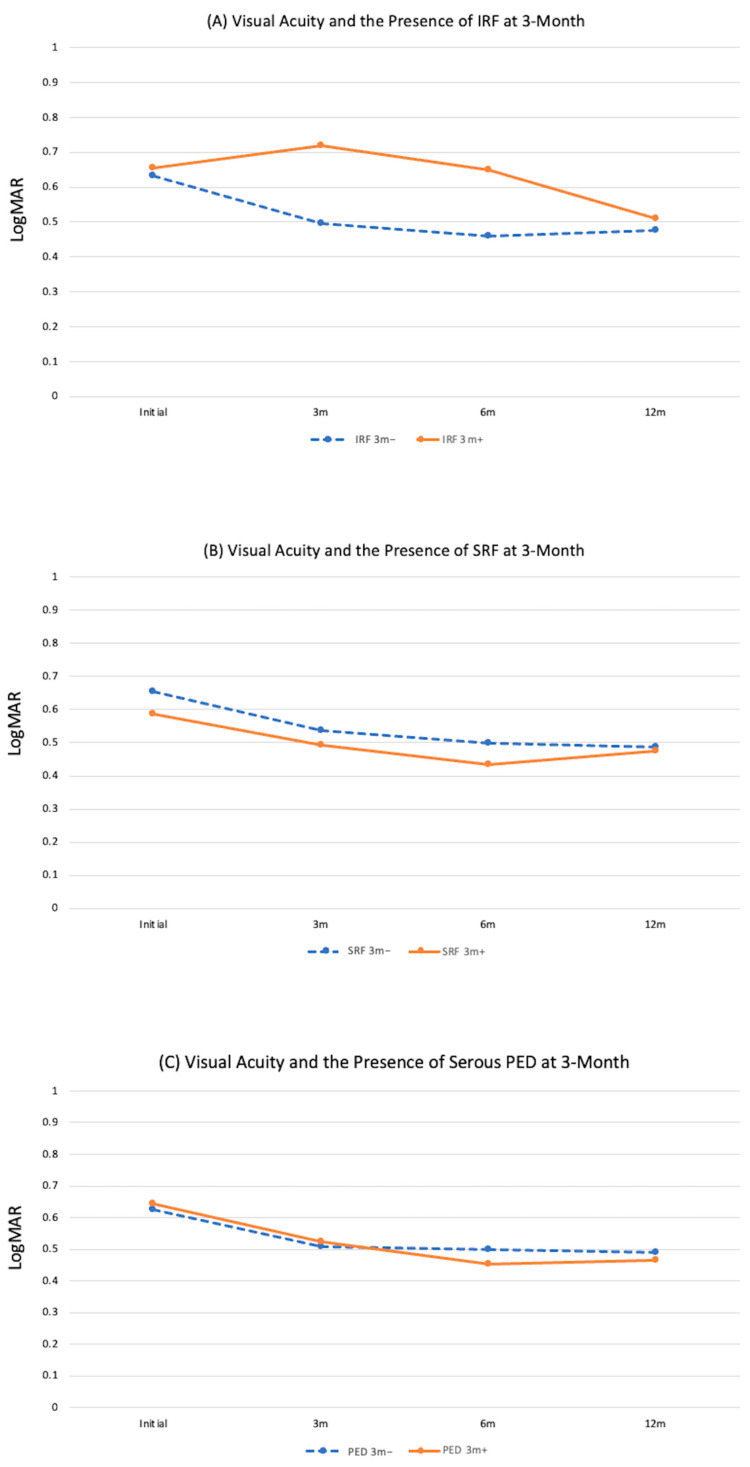
Visual acuity presented in logMAR at different time points before and after treatment in patients, according to the presence of fluid biomarkers at 3 months after the loading treatment. (**A**) Intraretinal fluid (IRF), (**B**) subretinal fluid (SRF), (**C**) serous pigment epithelial detachment (PED).

Three months after treatment, fluid in the macula improved considerably, as the presence of IRF decreased from 19.8% to 8.8%, SRF decreased from 75.8% to 20.9%, and serous PED decreased 76.9% to 49.5%. Eyes with IRF had poorer vision than those without IRF at 3 months, though this was not statistically significant (*p* = 0.28). In addition, none of these fluid biomarkers were predictive for vision at 12 months (*p* > 0.05 for all; Figure 5).

## 4. Discussion

PCV has different characteristics among different ethnicities, its incidence, clinical presentation, and treatment response varies. Although many treatment regimens and follow-up protocols for PCV and nAMD are similar, PCV in Asian population resembles pachychoroid spectrum disorders. In many clinical trials, the treatment goal for both nAMD and PCV was to achieve anatomical dryness without fluid in the intraretinal, subretinal, or sub-RPE layers [18,25,26,27]. Since fluid and hemorrhage are surrogates of disease activity, treatment decisions after the loading phase were largely based on the dryness of the retina [29,30,37]. Based on previous post hoc analyses of large clinical trials for nAMD and real-world experiences in nAMD treatment, we realized that the presence of IRF and serous PED leads to poor visual prognosis, whereas SRF seems to have some protective effects for VA [31,32,33].

The present study showed that SRF at baseline or any time point did not result in better visual PCV prognosis. Eyes without SRF tended to have better vision than those with SRF during the 12-month follow-up period, although no significant differences were observed between the two groups. This seems to contradict the findings that showed the presence of SRF had a protective role for VA in nAMD [31,33]. SRF has been postulated to contain nutrients and nourish the overlying outer retina. If there are defects in the RPE under the SRF, the SRF could serve as a medium for nutrition and metabolic exchange, and surrounding healthy RPE could aid in this process. Once the SRF is absorbed, however, the outer retina that is in direct contact with the diseased RPE may not receive adequate metabolic support, which would result in poor vision. In addition, eyes with nAMD that progressed to geographic atrophy were usually free from SRF, although their vision was very poor [38,39]. This may explain why SRF is not protective for visual function. However, the incidence of geographic atrophy was much lower in PCV than in nAMD [40]. Even in eyes without geographic atrophy, the function of RPE may differ between nAMD and PCV patients. For eyes with functioning RPE, the SRF fails to serve as a protective medium. Instead, SRF results in worse vision than a dry retina, as seen in cases of macula-off retinal detachment or chronic central serous chorioretinopathy. These observations may explain the different effects of SRF on VA between nAMD and PCV. Previous studies showed that visual deterioration usually happens several months after the appearance of SRF. There may be insidious onset of visual deterioration in cases of long-standing SRF where the SRF was not initially considered damaging [41,42]. Based on our results, we suggest that SRF should not be regarded as a protective sign and should be aggressively treated in PCV.

The presence of IRF has long been considered to cause poor visual outcomes in nAMD [31,33,41,43]. In the present study, we found that BCVA was significantly poorer in the group with IRF at 6 and 12 months after treatment. These results were consistent with findings for nAMD. Furthermore, eyes with IRF at baseline still had sustained visual deterioration after treatment, in contrast to sustained visual improvement in eyes without IRF at baseline. There has been debate on whether IRF and intraretinal cysts (IRCs) are different entities; the former arises from the accumulation of fluid, and the latter results from a degenerative process with the loss of retinal tissue [37]. In this study, we did not differentiate between fluid accumulation and degenerative cysts because many were indistinguishable in OCT. Therefore, we believe that the poor treatment effects in eyes with IRF at baseline may be partially due to the fact that degenerative cysts were already present. At baseline, the proportion of IRF was 19.8%, which declined to 8.8% and 13.2% at 3 and 6 months after treatment, respectively. Therefore, we still suggest aggressive treatment for fresh cases of PCV with IRF. However, if persistent IRCs without changes are observed after sustained treatment, it may be regarded as an inactive lesion and an extension of the treatment interval can be considered. Few patients in our cohort developed new cases of IRF during the treatment period. Those who developed IRF did encounter a decline in vision, and aggressive treatment was appropriate due to the reactivity of the disease.

The occurrence of serous PED preceded IRF accumulation and thus had a negative impact on vision in nAMD [34], and the improvement in vision was also limited even after treatment in eyes with serous PED [37]. In the present study, we found that PCV eyes with serous PED had poorer vision at baseline. Vision could improve after treatment, although there was a tendency for poorer final vision. The proportion of serous PED was as high as 76.9% at baseline in our study, which declined to 38.5% at 12 months after treatment. The reported prevalence of PED varied in other studies, ranging from 55% to 100% for PCV patients, including all patients with serous, hemorrhagic, and fibrotic PEDs [44,45].

The fluid biomarkers at 3 months were less relevant to the visual prognosis of PCV patients compared with the fluid biomarkers at baseline. These results were slightly different to those reported by previous studies on nAMD [33]. This may be due to essential disease activities and status. Additionally, this may indicate that at 3 months after treatment, separate markers (such as fluid accumulation in different compartments) may not be a good surrogate for VA outcome, as the disease is mostly still affected by the treatment and is therefore relatively suppressed at this time. Furthermore, the fluid may have either newly emerged or merely be residual. In our cohort, most fluids were residual fluids, which complicates their predictive value for visual prognosis.

Subfoveal hemorrhage secondary to choroidal neovascularization is associated with poor VA, and progressive VA loss was observed in AMD-related subfoveal hemorrhage without treatment [46,47]. Pneumatic displacement, intravitreal anti-VEGF injection, or recombinant tissue plasminogen activator (rTPA) injection were proposed as treatments for subfoveal hemorrhages, but they showed variable outcomes. Although intravitreal anti-VEGF injection improved vision after treatment, VA prognosis was not guaranteed as in those without subfoveal hemorrhages [48]. Our results showed a similar trend, with an improvement in VA after treatment in both groups with and without subfoveal hemorrhage. However, the VA of the group with initial hemorrhage was worse at all time points (*p* < 0.05).

This study was constrained by its retrospective design, which inherently limits the ability to establish causal relationships. Furthermore, the treatment protocols following the loading phases were not uniformly defined for all patients but rather adhered to the prevailing regulations of the National Health Institution at the time, introducing variability in treatment approaches. Despite efforts to enroll all cases meeting the inclusion criteria, the study did not conduct a preliminary sample size calculation, potentially impacting the statistical power of the analysis [49]. Additionally, the OCT model utilized lacked the advanced capabilities of swept-source OCT with enhanced depth of focus function, possibly leading to minor inaccuracies in individual thickness measurements. Another confounder was that during the study periods, OCT software version has several updates, although not necessarily affecting the targeted biomarkers, this might yield minimal inaccuracy. It is noteworthy that all cases underwent OCT imaging under mydriatic conditions, which may have marginally reduced the accuracy of choroidal thickness measurements [50]. However, this limitation is unlikely to significantly alter the core findings of the study. Notably, this investigation represents the inaugural endeavor to investigate the repercussions of fluid accumulation on visual outcomes in PCV patients. In the future, we could conduct a prospective study based on our current data, adding other visual function tests, such as microperimetry, for more thorough evidence. In summary, the findings suggest that all types of fluid accumulation exert a detrimental effect on visual acuity and necessitate prompt and aggressive management in the context of PCV.

## Data Availability

Data is available upon request (Yi-Ting Hsieh).
